# Black men’s awareness of peripheral artery disease and acceptability of screening in barbershops: a qualitative analysis

**DOI:** 10.1186/s12889-022-14648-x

**Published:** 2023-01-06

**Authors:** Tyler Coy, Ellen Brinza, Sarah DeLozier, Heather L. Gornik, Allison R. Webel, Christopher T. Longenecker, Khendi T. White Solaru

**Affiliations:** 1 Division of Cardiovascular Medicine and Harrington Heart and Vascular Institute, University Hospitals, Cleveland, OH USA; 2 Department of Medicine, Case Western Reserve University, Cleveland, OH USA; 3 Department of Internal Medicine, University of Colorado, Aurora, CO USA; 4 Clinical Research Center, University Hospitals, Cleveland, OH USA; 5 University of Washington School of Nursing, Seattle, WA USA; 6 School of Medicine and Department of Global Health, University of Washington, Seattle, WA USA

**Keywords:** Peripheral artery disease, Hypertension, Cardiovascular disease, Social determinants of health, Race, Racism, Health equity, Qualitative analysis, Community-based research

## Abstract

**Introduction:**

Peripheral artery disease (PAD) disproportionately burdens Black Americans, particularly Black men. Despite the significant prevalence and high rate of associated morbidity and mortality, awareness of and treatment initiation for PAD remains low in this demographic group. Given the well-established social cohesion among barbershops frequently attended by Black men, barbershops may be ideal settings for health screening and education to improve awareness, early detection, and treatment initiation of PAD among Black men.

**Methods:**

A qualitative study involving 1:1 participant interviews in Cleveland, Ohio assessed perspectives of Black men about barbershop-based screening and education about PAD. Inductive thematic analysis was performed to derive themes directly from the data to reflect perceived PAD awareness and acceptability of screening in a barbershop setting.

**Results:**

Twenty-eight African American/Black, non-Hispanic men completed a qualitative interview for this analysis. Mean age was 59.3 ± 11.2 years and 93% of participants resided in socioeconomically disadvantaged zip codes. Several themes emerged indicating increased awareness of PAD and acceptability of barbershop-based screenings for PAD, advocacy for systemic changes to improve the health of the community, and a desire among participants to increase knowledge about cardiovascular disease.

**Conclusions:**

Participants were overwhelmingly accepting of PAD screenings and reported increased awareness of PAD and propensity to seek healthcare due to engagement in the study. Participants provided insight into barriers and facilitators of health and healthcare-seeking behavior, as well as into the community and the barbershop as an institution. Additional research is needed to explore the perspectives of additional stakeholders and to translate community-based screenings into treatment initiation.

**Supplementary Information:**

The online version contains supplementary material available at 10.1186/s12889-022-14648-x.

## Background

Peripheral artery disease (PAD) is a morbid form of cardiovascular disease (CVD), most commonly referring to obstruction of the lower extremity arteries. Disease ranges in severity from asymptomatic or intermittent claudication to severe, limb-threatening disease known as chronic limb threatening ischemia (CLTI) [1, 2]. A diagnosis of PAD confers increased morbidity and mortality due to increased risk of myocardial infarction, ischemic stroke, limb loss, and death due to CVD. It is estimated to have contributed to roughly 500,000 years lived with disability and 1,000,000 years of life lost globally in 2019 [3]. Individuals living with PAD report poorer quality of life due to pain and discomfort, limited ability to engage in physical activity, depressive symptoms, and feelings of hopelessness [4]. Additionally, the disease and its associated morbidity is costly; it is estimated that US adults with PAD have higher healthcare-related expenditures compared to the general population [5]. Despite these poor outcomes, only 25% of Americans are familiar with PAD – the lowest recognition of all the CVD categories [6, 7].

Globally, the data for PAD prevalence among different ethnic and racial groups are limited; however, it is known that high-income countries such as the United States and the United Kingdom have higher rates of PAD compared with low- and middle-income countries (7.4% prevalence of PAD in high-income countries vs. 5.1% in low- and middle-income countries) [8, 9]. Further, Black men in the United States have the highest rates of PAD, with an estimated lifetime risk of developing PAD of 30% among Black Americans compared to only 20% among non-Hispanic white Americans [10]. Yet, Black Americans have the lowest reported awareness of PAD compared to other racial or ethnic groups with only 6% of Black Americans being aware of the morbid consequences of PAD [11]. Therefore, early detection and appropriate intervention for PAD among Black men is essential for reducing health disparities.

PAD is often underdiagnosed and undertreated, especially among Black Americans who present with more severe disease and experience worse health outcomes [12–15]. Black men are less likely to be treated for PAD or its predisposing conditions (e.g., smoking, hypertension, hyperlipidemia), less likely to undergo revascularization as limb salvage therapy for CLTI, more likely to experience lower limb amputation, and ultimately more likely to die from cardiovascular events [16–22]. These adverse health outcomes are associated with social and economic burden to both the individual and society [23, 24].

Race is often conceptualized as an independent risk factor for disease; however, emerging scholarship validates race as a mediator of structural inequalities resulting from racist policies [25]. Systemic racism reinforces policies and practices that operate within and across institutions to reinforce segregation and disadvantage people of color, namely Black Americans. For example, redlining, a practice whereby the federal government sponsored neighborhood rating of desirability to inform home lending, led to segregation by race and differences in neighborhood quality, community conditions, and racial differences in socioeconomic status [26–28]. To this day, living in historically redlined and underinvested neighborhoods is associated with adverse health conditions [26]. Inequities in housing, income, education level, and access to quality healthcare, resulting in part from systemic racism [29], can disadvantage underserved populations such as Black Americans, complicating both prevention and management of CVD, including PAD [19, 30, 31].

At the interpersonal level, Black Americans experience racism and discrimination in various settings, including in healthcare. Although well-intentioned, most healthcare providers hold negative implicit biases toward people of color, which may negatively impact patient-provider interactions, treatment, and health outcomes [32]. Further, historical exploitation and injustices against Black Americans in medicine and research (e.g., The U.S. Public Health Service Syphilis Study at Tuskegee) compound distrust of public health and medical institutions [33], which may impact an individual’s propensity to engage with healthcare or research. Beyond physical access to healthcare, “access” may be further envisioned to include mitigation of providers’ implicit biases, and validation of Black Americans’ historical mistrust of healthcare to build trust to prevent, detect, and treat CVD.

Barbershops are cultural institutions among Black men and allow for open discussion about various topics. Previous studies demonstrate the effectiveness of barbershop-based education and screening in increasing knowledge about various health topics, promoting healthy behaviors and adherence to treatment, and improving blood pressure management [34–39]. Given that Black men are less likely than other racial groups or Black women to access healthcare [40], and that barbershops are frequently visited among Black men and ideal for ethnographic exploration [34, 41], barbershops may be an ideal setting to improve PAD awareness among Black men. This qualitative study aimed to explore the acceptability of performing PAD screenings in the barbershop setting. To our knowledge, this is the first study to screen for PAD in a barbershop setting and conduct an in-depth examination of participants’ perspectives.

## Methods

### Study design

This qualitative sub-study was part of a larger pilot study approved by the University Hospitals Cleveland Medical Center Institutional Review Board [42]. The aims of this sub-study were: 1) assess the acceptability of barbershop-based screening for hypertension and PAD, and 2) assess perceived awareness of PAD among Black men. The design and methods of the pilot study have been published elsewhere. In brief, both a manual Doppler and an automated device were used for measurement of the ankle-brachial index (ABI) to screen for PAD, in accordance with the published standards [42]. To explore the perceived acceptability of the study intervention, our team performed thematic analysis of interviews conducted at participants’ final study visit.

### Study population and recruitment

The pilot study took place in two Black-owned barbershops in Cleveland, Ohio between June and December 2020. No relationship between study staff and participants had been established prior to study commencement. Barbershop patrons were approached (*n* = 98) by a study team member for eligibility screening to obtain a convenience sample. Eligible patrons (*n* = 74) identified as African American or Black and non-Hispanic men, were between the ages of 40 and 89, and regularly attended the barbershop. Thirty-seven eligible participants agreed to participate in the pilot study and signed written informed consent. Patrons who declined to participate did so primarily due to time constraints, not wanting to participate in research, or because they already knew their health status.

### Study visits

Study participants completed an initial screening visit and underwent blood pressure and PAD screening using an automated ABI device, followed by a visit 4–6 weeks later when ABI was obtained using the gold-standard Doppler-based methods [43]. Fingerstick cholesterol and glucose readings were also obtained at the second visit. Participants were invited to watch an educational video about PAD following the second visit. A final visit took place 4–6 weeks after the second visit where interviews were conducted. Participants were compensated $100 over the duration of the study, including $80 cash and a $20 restaurant gift card.

### Interview

Participants were invited to participate in an interview at the final visit. A qualitative interview guide (see Additional file 1) was developed to explore perspectives on different facets of the intervention. One-on-one interviews were conducted in a private area of the barbershop primarily by investigator TC (white male-identified) and lasted roughly 10–15 minutes. Investigator KWS (Black female-identified) also conducted a portion of the interviews. Investigators were mindful of racial and gender discordance, respectively, but this barrier was in part overcome by practicing cultural sensitivity, employing interpersonal skills, and demonstrating authenticity to build trust [44–46]. Probing questions were used throughout interviews to encourage expansion on responses. Audio files from interviews were recorded using unique identifiers to maintain confidentiality, uploaded to a secure server, and later manually transcribed verbatim by TC and EB (white female-identified). Transcriptions were uploaded to Dedoose, a secure, web-based program for qualitative data management and analysis [47].

### Qualitative analysis

An inductive thematic analysis was conducted using Braun and Clarke’s framework [48]. Research on the perceptions of Black Americans undergoing PAD screening and education – particularly in community-based settings – is virtually non-existent. As such, an inductive approach allowed for the data itself (i.e., the lived experiences of Black Americans) to inform the generation of theory. The codebook was developed using an inductive approach, with the codes directly derived from interpretations of the qualitative data itself [49]. Team members (TC, EB, KWS) independently read through all transcripts and engaged in reflexive journaling, identifying common patterns in the data that helped guide the formulation of a preliminary codebook. Meaningful, exemplar excerpts related to the study objectives were identified while reviewing the transcripts; these were used to create categories of data from which codes were developed. Coders (TC and EB) met regularly to discuss codes and definitions to ensure codes had explicit boundaries, were not interchangeable nor redundant [50], and tested the codebook using a representative sample of transcripts. KWS served as an external check on the research process to ensure credibility (i.e., trustworthiness) of the data by checking preliminary findings against raw data and personal observations during study administration [51]. Upon reaching consensus on the final codebook (see Additional file 2), coders independently coded all 28 transcripts. Coded excerpts were adjudicated at team meetings until 100% agreement was achieved. The final coded excerpts were reviewed to generate themes based on corresponding data. Proposed themes were adjudicated during team meetings until consensus was reached. If a theme was observed by only one coder, then data were reviewed to ensure themes were semantic or explicit rather than latent or interpretive [50]. Finally, once themes were adjudicated, the team read through each transcript to ensure the final themes were representative of the dataset. Quantitative data collected during study visits were reviewed for the purposes of triangulation [52].

## Results

A total of 37 participants were enrolled in the study, and 28 participants completed the final visit and interview. The remaining study participants were lost-to-follow-up. The average age of participants was 59.3 ± 11.2 years, and most did not hold a high school graduate degree (Table 1). Nearly all participants (93%) reside in a zip code in Cleveland, Ohio with socioeconomic needs greater than the national average, as determined by Dignity Health’s Community Needs Index (CNI) [53] (see Additional file 3). Quantitative results from the clinical measurements in the larger pilot study are published elsewhere [42], but a summary of the results as it relates to this sub-study is provided here: Of those who completed the final visit, a total of 4/28 (14%) had a clinically significant value (ABI ≤ 0.90), none of whom were previously aware of their PAD diagnosis. 19/28 participants (68%) had hypertension, defined as at least two values of systolic blood pressure of ≥130 and/or diastolic blood pressure of ≥80 on at least two separate visits [54]. 8/19 (42%) were unaware of their hypertension diagnosis prior to the study.

**Table 1:** Participant Demographics and Medical History

	Number (%)
Past medical history	
Hypertension (high blood pressure)	14 (50)
Peripheral artery disease (PAD)	0 (0)
Diabetes	5 (18)
Hyperlipidemia (high cholesterol)	7 (25)
Age	59.3 ± 11.2
Have you ever smoked cigarettes?	
Yes	22 (79)
No	6 (21)
Former user	5 (18)
Current user	16 (57)
How many years did/have you smoked?	27.6 ± 18.0
How many packs of cigarettes did/do you smoke per day?	0.7 ± 0.6
Highest education level?	
Not a high school graduate	12 (43)
High school graduate or GED	8 (29)
Some college/associate’s degree	7 (25)
Bachelor’s degree	0 (0)
Graduate or professional degree	1 (4)
Have you seen a primary care doctor or other provider (e.g., nurse practitioner) in the past year?	
Yes	19 (68)
No	9 (32)
Have you seen a cardiologist or vascular specialist in the past year?	
Yes	6 (21)
No	22 (79)
Have you been screened for peripheral arterial disease (PAD) using an Ankle Brachial Index (ABI) in the past?	
Yes	4 (14)
No	24 (86)
Were you diagnosed with PAD?	
Yes	0 (0)
No	4 (100)
Do you have health insurance?	
Yes	22 (79)
No	6 (21)
How often do you come to this barbershop now?	
More than twice a month	13 (46)
Once or twice a month	13 (46)
Less than once a month	2 (7)

Seven main themes emerged from the data (Table 2).

**Table 2:** Themes identified through inductive analysis and exemplar quotes

*Theme*	*Exemplar Quote*
**Motivation to seek healthcare among Black men exists, yet barriers such as fear, trust, and access persist.** Participants described numerous barriers that impede their ability or the ability of members of their community to seek care from the healthcare system, often leading to negative associations about healthcare. These barriers included time, cost of visits, unreliable transportation to healthcare facilities, potential psychological effects of bad news, feelings of fear, or a lack of health insurance. While barriers exist, participants expressed motivation for health and often described changes to their healthcare interactions ascribed to the study.	*“We need more of this. This is a beautiful start. We… need more awareness programs for everybody to reach out. And make it more open to people so they feel more comfortable going to see their doctors. Hospitals are crowded right now, they booked up. And a lot of people scared of that medical fee. So when you get somebody to come out in the neighborhood and provide you with a blood test or something like that or blood pressure, it helps… Honestly, if I’m not sick or injured, I don’t do usually check-ups because… I don’t know. I don’t like going to the doctor. I mean that’s just the most honesty I can be. It’s like because sometimes you just don’t want to feel, I mean find out if something is wrong or not… You don’t want to search out that something wrong when you don’t feel like something wrong.”* – *Participant 36 (Age: 48)*
**Knowledge about health status is empowering, reassuring, and precipitates health behavior change** The knowledge received about individual health status seemed to alleviate worry, stress, or anxiety about health and often facilitated or helped to promote self-efficacy and empower lifestyle change(s) and increased interactions with the healthcare system.	*“I understand what’s going on with my body more. You know what I’m saying? Sometimes when you’re always working, you know and back and forth on different jobs and stuff like that, you don’t pay any attention to your health. Now, actually looking at the length and seeing that this could be actually life-threatening… it’s time to really look in the mirror and change your life a little bit... I might be able to turn this around. Yeah, but it’s gonna take a lot of dedication to myself. And I do love myself so.” – Participant 32 (Age: 47)*
**Community-based education intervention enhanced knowledge of cardiovascular disease** Participants often reported how the study helped to provide enhanced information about chronic disease, specifically PAD and hypertension, and often promoted further learning about health.	*“Once I found out what PAD is and so on and so forth, you know it deals with diabetes, hypertension, high cholesterol, yeah, that’s the entire black community, so yes. Yeah it went together. It had a good correlation.” – Participant 9 (Age: 46)*
**Convenience of accessing health care resources in the barbershop** Accessibility and comfort of the barbershop helps overcome barriers to seeking care, such as mistrust or fear of the healthcare system, time burden of regular visits, and socioeconomic barriers.	*“I was glad it was here and it was easily accessible. I didn’t have to go to the hospital and pay for parking, and actually it was right in line with what I was doing… I think it’d be a good idea if y’all could do something on a similar scale for other healthcare needs for guys in the community, ‘cause people just come through the barbershop and with their regular schedule, but it’s kinda hard … to schedule in a doctor’s appointment because it’s gonna take much more time because you gotta park, get there, wait… But if it’s already in line with what I’m doing anyway, I can swing right through here… and just paying for everything… And then just having time, because most people in the community gotta work as many hours as possible to make ends meet, so I don’t have an extra two hours to go there, but I might have enough time to slide through ‘cause I gotta get a haircut for where I’m working at, so I gotta keep myself particularly groomed, so this is like a part of work. You know what I mean. So, but I don’t have time to go to the doctor.” – Participant 9 (Age: 46)*
**Despite disparities, participants advocate for health of community** Participants regularly mentioned knowledge, either pre-existing or ascribed to the study, about health disparities in the black community and propensity to seek healthcare. Although disparities persist, participants seem to serve as ambassadors of the health of others in the community.	*“Black people we do a lot of high cholesterol, high blood pressure. It brings the worry next to us to see that it is an epidemic – I mean not an epidemic, but it is a serious matter. And it does affect us more than it affects a lot of others. Racist. Okay? It is ‘cause we do suffer, I don’t know if it’s our way of cooking, our way of living, but it definitely affect us because we always have high blood pressure. Diabetes runs heavily amongst African Americans. So yeah, it’s very good because we should be aware. Some people just take it for granted. And it’s beautiful for you to take it out to our neighborhoods and let anybody just find out about it…” – Participant 36 (Age: 48)*
**Positive study feedback provided by participants** Participants expressed an overall positive experience with the study, including interactions with the study team, satisfaction with health screenings, convenience, and simplification of material. This facilitated engagement in the study and general satisfaction. The large majority said they would participate again, and frequently expressed wanting to see a similar model expanded to address health needs of the community.	*“I’m a person of showmanship. And if you got the right people running it in the barbershop or in the neighborhood, then just in that alone would bring me back. There was no stress. It was a comfortable meeting. You talked to me, not at me. Saying this is what you’re supposed to do – you didn’t do none of that. You made every visit very comfortable.” - Participant 32 (Age: 47)*
**Participation driven by both a desire to know health status and altruism** The number one reason that participants listed as their reason to participate in the study was to gain knowledge about their health status. Participation in this study was a convenient and accessible way of learning about health status. Participants also mentioned participating not only for the benefit of the community, but also for the sake of research or the medical community. Finally, while compensation was helpful, it was a secondary gain, or “added bonus.”	*“Again, I get this done. I just did this to show some other people, you know, you should do it… So that does push people towards to have that benefit done. You got a role model figure that’s there – ‘that role model’s gonna get me there.’”* – *Participant 3 (Age: 61)*

### Motivation to seek healthcare among black men exists, yet barriers such as fear, trust, and access persist

A continuum of the propensity to engage with the healthcare system was identified among participants. Some described yielding autonomy to providers, while others avoided engaging in healthcare interactions altogether. Notable barriers to accessing care were fear and/or discomfort associated with the detection of clinically significant news.

*“A lot of black males are frightened to get themselves checked out. They’d rather jump out of an airplane at 450,000 feet in the air, knowing that they have an operable parachute.” – Participant 15 (Age: 58).*

The physical presence of a healthcare intervention in the community seemed to help overcome barriers associated with low propensity to engage with the healthcare system.

*“If y’all out in the community, that might show somebody else that they shouldn’t be afraid to get themselves checked out, because a lot of people don’t come to the doctor… they’re scared.” – Participant 1 (Age: 56).*

Several participants conceptualized healthcare as primarily for emergencies, but some expressed that engaging with the study promoted follow-up with a healthcare provider. One participant without health insurance noted:

*“You guys let me know now, I have to go see [a] doctor because if I’m healthy too, I need to see doctor.” – Participant 13 (Age: 55).*

While participants expressed motivation to engage with healthcare, they also described many socioeconomic challenges in addition to barriers to seeking care, including lack of trust in the healthcare system and scheduling or cost logistics (including lack of health insurance). Five participants (18%) self-reported experiencing discrimination previously while seeking healthcare. Additionally, only 19 participants (68%) have regular access to the internet, and seven participants (25%) reported having trouble getting food and groceries during the COVID-19 pandemic.

*“Most people in the community gotta work as many hours as possible to make ends meet, so I don’t have an extra two hours to go there, but I might have enough time to slide through ‘cause I gotta get a haircut for where I’m working at… but I don’t have time to go to the doctor.” – Participant 9 (Age: 46).*

### Knowledge about health status is empowering, reassuring, and precipitates health behavior change

Fourteen participants (50%) reported that they do not have a regular healthcare provider and only 19 (68%) participants reported having seen a primary care provider in the past year. This may have been exacerbated during the COVID-19 pandemic, given that 12 participants (43%) reported resisting seeking medical care or not going to the doctor for fear of contracting the virus, or that 7 participants (25%) reported their insurance being impacted by the pandemic. Many participants who do not regularly receive primary care noted that engaging with the study and learning about their health status was reassuring and increased accountability.

*“I worry because I hadn’t been checking on me like I should have. And I had a better opportunity to be seeing what was wrong and what was right… it helped me.” – Participant 16 (Age: 83).*

Participants with clinically significant test results described how the results prompted reflection about health habits and promoted self-efficacy.

*“[The results] made me realize how serious it is to take care of my body. And I’m getting more serious about it… I’m going to do a little bit more stuff to improve my… health. Already eating better, start walking more. Exercising a little more. And keep following up…[with] screenings and more check-ups.” – Participant 34 (Age: 65).*

Similarly, one participant who was diagnosed with PAD as a result of participating in this study reported new motivation to quit smoking as a result of knowledge gained from the study.

*“Now I see I gotta stop smoking. I see I gotta do a little bit different…healthy eating habits… Now that I’ve been… listening to the professionals talk about it… I don’t want to wake up one morning like, ‘Oh my god, my leg gotta come off tomorrow?’ That would be devastating.” – Participant 32 (Age: 47).*

### Community-based education intervention enhanced knowledge of cardiovascular disease

Across the participant population, awareness of PAD was generally low at the beginning of the study. Participants reported that information provided in the study helped to facilitate enhanced knowledge about PAD and chronic disease.

*“Once I found out what PAD is… It deals with diabetes, hypertension, high cholesterol…, that’s the entire black community… It had a good correlation.” – Participant 9 (Age: 46).*

Similarly, after learning about the adverse outcomes associated with tobacco use, including increased risk of hypertension and PAD, one participant highlighted his gratitude for quitting.

*“[The study] gave me more of an insight…on how [PAD] affects…the Black community. By smoking and the high blood pressure just let me know that I’m very grateful that I quit smoking.” – Participant 8 (Age: 63).*

While there was a general consensus that the study facilitated enhanced medical knowledge among participants, there were notable opportunities for improvement. Importantly, some participants expressed that medical jargon occasionally stood in the way of knowledge gain, and some content was incongruent with prerequisite health literacy.

*“Not saying all the complicated medical terms [makes it more understandable] … if you’re going to talk to a patient, break it down to layman’s terms.” – Participant 36 (Age: 48).*

These findings highlight the need to modify future educational materials, tailoring them to individuals of differing levels of healthcare literacy.

### Convenience of accessing health care resources in the barbershop

Participants described the barbershop as a community hub for Black men that serves primarily as a gathering place. Several participants reported attending their respective barbershop for many years, and nearly all participants (92%) reported attending the barbershop at least once monthly.

*“I’ve been coming down here since I was kid. So about the last forty some odd years, everybody gonna come to the barbershop for a haircut. They come for everything else. The haircut just happen to happen.” – Participant 9 (Age: 46).*

Several participants described the compounding economic and healthcare access challenges that the Black community faces and spoke about the utility of health screenings in the barbershop setting.

*“Some Black people here…never see the doctor because they don’t have insurance, they don’t have a car, they don’t have nothing. But if you say, “Go to barbershop,” they can walk, come to the barbershop.” – Participant 13 (Age: 55).*

### Despite disparities, participants advocate for health of community

Several participants expressed the influence role models have in shaping community health. All participants expressed satisfaction with health screenings and most encouraged the study team to continue initiatives for the betterment of the community.

*“If there was a need, yes [I would participate again] ...Anything in reference to help… myself as well as others.” – Participant 12 (Age: 55).*

### Positive study feedback provided by participants

Most participants described positive experiences and generally denied discomfort or burden with study procedures such as the ABI test, although some noted the need for a larger space in the barbershop for study procedures.

*“Oh yeah, that [ABI procedure] didn’t bother me. I was telling my girl what y’all had done, you know. And I said, I just laid there and they checked both legs and checked both arms. And it was alright. It didn’t take long… I felt comfortable. I got comfortable when y’all was here.” –Participant 33 (Age: 62).*

Many equated their positive experience with receiving good news about their health status.

*“I wouldn’t have felt good if you were to tell me my blood pressure was high. I think that would’ve been a downfall on me.” – Participant 35 (Age: 56).*

### Participation driven by both a desire to know health status and altruism

Most participants reported that the primary motivation to participate and engage in the study was a desire to know their health status.

*“I’d say [compensation was] appropriate, plus the knowledge you get is more valuable than money. You know you use money, but you get to know about what’s going on inside you. And that’s more valuable.” – Participant 34 (Age: 65).*

While compensation was helpful to some, particularly during the COVID-19 pandemic, when asked whether the compensation provided by the study was appropriate, participants described their concerns for the community.

*“…You guys are helping the community, and I’m hand in hand with you guys. Some people don’t have no doctor or nothing like that.” – Participant 1 (Age: 56).*

## Discussion

Peripheral artery disease (PAD) disproportionately burdens Black Americans [14, 20], heightening the risk of poor health outcomes, including reduced quality of life, major cardiovascular events (i.e., heart attack, stroke), and limb amputation [10, 55]. The seven themes identified in this qualitative study demonstrate that barbershop-based PAD screenings are perceived to be acceptable among Black men, and may help facilitate early disease detection.

While recognition for the importance of community-based participatory research interventions exists [56], few have explored perceptions among patrons in barbershop interventions. Our first aim was to assess the acceptability of barbershop-based screening for PAD. The overwhelmingly positive feedback among study participants supports the acceptability of barbershop-based PAD screenings. This is notable, considering that ABI testing, just one component of our study, requires more time than health screenings employed in other barbershop-based interventions, such as blood pressure measurements and glucose checks [56, 57]. Participants noted several factors important for successful implementation, including a large, private space to conduct ABI testing and information delivery tailored to individual health literacy. Further, community was identified as a beacon of health among our study population, and existing social cohesion within barbershops seemed to facilitate enhanced participant engagement, consistent with prior literature [58]. Both the physical presence of a health intervention in a trustworthy location and the buy-in from barbers and community members as role models may have helped to overcome barriers to healthcare-seeking behavior associated with historic mistrust or fear. The pervasive support expressed by participants contrasts with previous findings, where only 11.8% of Black men reported feeling comfortable undergoing barbershop-based education and screenings via trained barbers [59], although screenings in this study were conducted by healthcare professionals. While prior studies demonstrate improved disease management with barbers as health educators [37, 38], future research should explore participants’ acceptance of this model involving healthcare professionals in the barbershop setting. Further, while only one participant explicitly mentioned the word “racist,” the various socioeconomic challenges described by participants [28], disparities in rates of PAD and its associated impacts on, and low propensity to engage with preventative healthcare [19, 60], are likely associated with systemic racism. Similarly, while participants described fear as a barrier to seeking healthcare, it was unclear whether fear is associated with masculinity, or perceived or experienced discrimination in healthcare. Given that interpersonal and systemic racism persist, future research should more intentionally and explicitly explore participants’ perceptions as it relates to interpersonal and systemic racism.

Our second aim was to assess participants’ perceived awareness of PAD. Most participants stated they had minimal awareness of PAD prior to the intervention and reported aspirational lifestyle modifications after learning about PAD, including smoking cessation. This is noteworthy given the high prevalence (57%) of smoking among our study population. Even for participants with normal clinical values, the knowledge gained about PAD served as a catalyst for raising awareness in the community about the disease, being mindful of lifestyle choices, and engaging in preventative care. These findings highlight the need for additional community-based PAD education and screening initiatives. Consistent with prior studies [61, 62], participants frequently described reserving interactions with the healthcare system for emergency situations. This study seemed to expand conceptualization of healthcare to include preventative care, evidenced by participants’ reported increased engagement in preventative care. Additional community-based initiatives that highlight the importance of preventative care and exposure to health-promoting norms may help improve health outcomes among this population [63, 64], including early detection of PAD. However, while building trust and delivering culturally competent education may help to detect and initiate treatment for PAD, an onus should also be placed on healthcare, public health institutions, and governments to address individuals’ social needs and improve community conditions to correct historical injustices that contribute to disparate CVD outcomes.

Lessons gleaned from this study may inform future community-based interventions for CVD. First, participants expressed that barbershop-based screening helped overcome barriers to seeking care, including fear or a lack of trust associated with the healthcare system, socioeconomic challenges, and a low propensity to access preventative care – barriers previously described as unique challenges for Black men [60, 63, 65]. Future research should explore factors that negatively affect Black Americans’ experiences with or perceptions of healthcare, including fear and mistrust potentially associated with providers’ implicit biases or historical injustices toward Black Americans [30, 32, 33]. As such, community members should be involved in designing and implementing community-based research studies. Finally, although individual health was highly valued among our study population, considerable external determinants of health beyond individuals’ control were noted throughout. Therefore, efforts to prevent and address individuals’ social needs may prove to be promising in mitigating health disparities. Further efforts should be made to understand and address social determinants of health and to eradicate barriers that stand in the way of the cardiovascular health of Black men.

## Limitations

There were several limitations to our study. Given that we used an inductive approach to explore semantic themes strictly representative of the entire dataset, it is possible that a different or larger sample of individuals may have allowed for better exploration of minor, latent themes. Additionally, it is possible that participants were not entirely forthcoming or that the racial discordance between the interviewer (TC) or gender discordance between the interviewer (KWS) and participants impacted their responses, as well as initial recruitment in the study [45]. As a means to overcome this, KWS – who shared racial concordance with participants – served as a primary contact, led study recruitment, and oversaw all study activities while TC (male-identified) conducted most of the interviews and allowed for fluid conversation in gender concordant contexts. The qualitative researchers (TC and EB) acknowledge inevitably held implicit biases about the study population and were committed throughout both study visits and analysis to confronting, mitigating, and discussing these biases. Given that those who participated in an interview attended multiple study visits and completed the study, it is possible that the views and experiences synthesized do not represent those who may not have valued this method of screening, or those who were lost to follow-up or unwilling to consent to the feasibility study. Therefore, our results may be favorably biased and may not be generalizable to all participants in the study or to the greater population. Finally, participants did not provide feedback on the themes identified, and we did not include barber or other stakeholder responses as a measure of acceptability; future research should explore the perspectives of these key stakeholders.

## Conclusions

This qualitative study highlights the perspectives of Black men who participated in a barbershop-based research study involving screening for and education about PAD. Findings support the acceptability of implementing barbershop-based PAD education and screening. Participants demonstrated increased knowledge of PAD and provided insights into both barriers and facilitators of health and healthcare-seeking behavior, as well as the barbershop as a cultural institution. These insights may help guide the implementation of future barbershop-based interventions, as well as other community-based screening and education initiatives.

## Supplementary Information

**Additional file 1.** HyPe The Cure Exit Interview Guide.

**Additional file 2.** HyPe The Cure Final Adjudicated Codebook and Definitions.

**Additional file 3.** HyPe The Cure Participant Community Needs Index (CNI)* Score by Zip Code.

## Data Availability

The data that support the findings of this study are not openly available due to reasons of human subjects protections but may be made available from the corresponding author upon reasonable request and approval from University Hospitals Cleveland Medical Center IRB.

## Abbreviations

PAD: Peripheral Artery Disease
CVD: Cardiovascular Disease
CLTI: Chronic Limb Threatening Ischemia
ABI: Ankle-Brachial Index
CNI: Community Needs Index

## References

[1] Gerhard-Herman MD, Gornik HL, Barrett C, Barshes NR, Corriere MA, Drachman DE, et al. AHA/ACC guideline on the management of patients with lower extremity peripheral artery disease: executive summary: a report of the American College of Cardiology/American Heart Association task force on clinical practice guidelines. Circulation. 2016;2017. 10.1161/CIR.0000000000000470. 10.1161/CIR.0000000000000470 PMC5479414 27840332

[2] Firnhaber JM, Powell CS. Lower extremity peripheral artery disease: diagnosis and treatment. Am Fam Physician. 2019. 10.1177/2047487313519344. 30874413

[3] Lin J, Chen Y, Jiang N, Li Z, Xu S. Burden of peripheral artery disease and its attributable risk factors in 204 countries and territories from 1990 to 2019. Front Cardiovasc Med. 2022. 10.3389/fcvm.2022.868370. 10.3389/fcvm.2022.868370 PMC9039520 35498034

[4] Wu A, Coresh J, Selvin E, Tanaka H, Heiss G, Hirsch AT, et al. Lower extremity peripheral artery disease and quality of life among older individuals in the community. J Am Heart Assoc. 2017. 10.1161/JAHA.116.004519. 10.1161/JAHA.116.004519 PMC5523635 28108464

[5] Scully RE, Arnaoutakis DJ, DeBord SA, Semel M, Nguyen LL. Estimated annual health care expenditures in individuals with peripheral arterial disease. J Vasc Surg. 2018. 10.1016/j.jvs.2017.06.102. 10.1016/j.jvs.2017.06.102 28847660

[6] Subherwal S, Patel MR, Kober L, Peterson ED, Bhatt DL, Gislason GH, et al. Peripheral artery disease is a coronary heart disease risk equivalent among both men and women: results from a nationwide study. Eur J Prev Cardiol. 2015. 10.1177/2047487313519344. 10.1177/2047487313519344 24398369

[7] Roger VL, Go AS, Lloyd-Jones DM, Adams RJ, Berry JD, Brown TM, et al. Heart disease and stroke statistics--2011 update: a report from the American Heart Association. Circulation. 2011. 10.1161/CIR.0b013e3182009701. 10.1161/CIR.0b013e3182009701 PMC4418670 21160056

[8] Song P, Fang Z, Wang H, Cai Y, Rahimi K, Zhu Y, et al. Global and regional prevalence, burden, and risk factors for carotid atherosclerosis: a systematic review, meta-analysis, and modelling study. Lancet Glob Health. 2020. 10.1016/S2214-109X(20)30117-0. 10.1016/S2214-109X(20)30117-0 32353319

[9] Aday AW, Matsushita K. Epidemiology of peripheral artery disease and polyvascular disease. Circ Res. 2021. 10.1161/CIRCRESAHA.121.318535. 10.1161/CIRCRESAHA.121.318535 PMC8202714 34110907

[10] Matsushita K, Sang Y, Ning H, Ballew SH, Chow EK, Grams ME, et al. Lifetime risk of lower-extremity peripheral artery disease defined by ankle-brachial index in the United States. J Am Heart Assoc. 2019. 10.1161/JAHA.119.012177. 10.1161/JAHA.119.012177 PMC6818002 31500474

[11] Fowkes FGR, Rudan D, Rudan I, Aboyans V, Deneberg J, McDermott MM, et al. Comparison of global estimates of prevalence and risk factors for peripheral artery disease in 2000 and 2010: a systematic review and analysis. Lancet. 2013. 10.1016/S0140-6736(13)61249-0. 10.1016/S0140-6736(13)61249-0 23915883

[12] Collins TC, Johnson M, Henderson W, Khuri SF, Daley J. Lower extremity nontraumatic amputation among veterans with peripheral arterial disease: is race an independent factor? Med Care. 2002. 10.1097/00005650-200201001-00012. 10.1097/00005650-200201001-00012 11789623

[13] Soden PA, Zettervall SL, Deery SE, Hughes K, Stoner MC, Goodney PP, et al. Black patients present with more severe vascular disease and a greater burden of risk factors than white patients at time of major vascular intervention. J Vasc Surg. 2018. 10.1016/j.jvs.2017.06.089. 10.1016/j.jvs.2017.06.089 PMC5794625 28951156

[14] Shu J, Santulli G. Update on peripheral artery disease: epidemiology and evidence-based facts. Atherosclerosis. 2018. 10.1016/j.atherosclerosis.2018.05.033. 10.1016/j.atherosclerosis.2018.05.033 PMC6113064 29843915

[15] Hughes K, Boyd C, Oyetunji T, Tran D, Chang D, Rose D, et al. Racial/ethnic disparities in revascularization for limb salvage: an analysis of the National Surgical Quality Improvement Program database. Vasc Endovasc Surg. 2014. 10.1177/1538574414543276. 10.1177/1538574414543276 25080452

[16] Collins TC, Slovut DP, Newton R Jr, Johnson WD, Larrivee S, Patterson J, et al. Ideal cardiovascular health and peripheral artery disease in African Americans: results from the Jackson heart study. Prev Med Rep. 2017. 10.1016/j.pmedr.2017.05.005. 10.1016/j.pmedr.2017.05.005 PMC5447374 28593118

[17] Huen KH, Chowdhury R, Shafii SM, Brewster LP, Arya S, Duwayri Y, et al. Smoking cessation is the least successful outcome of risk factor modification in uninsured patients with symptomatic peripheral arterial disease. Ann Vasc Surg. 2015. 10.1016/j.avsg.2014.09.014. 10.1016/j.avsg.2014.09.014 25286112

[18] Lipworth L, Fazio S, Kabagambe EK, Munro HM, Nwazue VC, Tarone RE, et al. A prospective study of statin use and mortality among 67,385 blacks and whites in the southeastern United States. Clin Epidemiol. 2013. 10.2147/CLEP.S53492. 10.2147/CLEP.S53492 PMC3872085 24379700

[19] Goyal A, Cooper HA, Aronow WS, Nagpal P, Yandrapalli S, Nabors C, et al. Use of statins for primary prevention: selection of risk threshold and implications across race and gender. Am J Med. 2018. 10.1016/j.amjmed.2018.05.029. 10.1016/j.amjmed.2018.05.029 29928863

[20] Carnethon MR, Pu J, Howard G, et al. Cardiovascular health in African Americans: a scientific statement from the American Heart Association. Circulation. 2017. 10.1161/CIR.0000000000000534. 10.1161/CIR.0000000000000534 29061565

[21] Allison MA, Ho E, Denenberg JO, Langer RD, Newman AB, Fabsitz RR (2007). Ethnic-specific prevalence of peripheral arterial disease in the United States. Am J Prev Med.

[22] Jones WS, Patel MR, Dai D, Subherwal S, Stafford J, Calhoun S, et al. Temporal trends and geographic variation of lower-extremity amputation in patients with peripheral artery disease: results from U.S. Medicare 2000-2008. J Am Coll Cardiol. 2012. 10.1016/j.jacc.2012.08.983. 10.1016/j.jacc.2012.08.983 PMC3918457 23103040

[23] Duff S, Mafilios MS, Bhounsule P, Hasegawa JT. The burden of critical limb ischemia: a review of recent literature. Vasc Health Risk Manag. 2019. 10.2147/VHRM.S209241. 10.2147/VHRM.S209241 PMC6617560 31308682

[24] Marrett E, DiBonaventura MD, Zhang Q. Burden of peripheral arterial disease in Europe and the United States: a patient survey. Health Qual Life Outcomes. 2013. 10.1186/1477-7525-11-175. 10.1186/1477-7525-11-175 PMC3854518 24148832

[25] Cerdeña JP, Plaisime MV, Tsai J. From race-based to race-conscious medicine: how anti-racist uprisings call us to act. Lancet. 2020. 10.1016/S0140-6736(20)32076-6. 10.1016/S0140-6736(20)32076-6 PMC7544456 33038972

[26] Lee EK, Donley G, Ciesielski TH, Gill I, Yamoah O, Roche A, et al. Health outcomes in redlined versus non-redlined neighborhoods: a systematic review and meta-analysis. Soc Sci Med. 2022. 10.1016/j.socscimed.2021.114. 10.1016/j.socscimed.2021.114696 34995988

[27] Williams DR, Sternthal M. Understanding racial-ethnic disparities in health: sociological contributions. J Health Soc Behav. 2010. 10.1177/0022146510383838. 10.1177/0022146510383838 PMC3468327 20943580

[28] Williams DR, Collins C. Racial residential segregation: A fundamental cause of racial disparities in health. Public Health Rep. 2001. 10.1093/phr/116.5.404. 10.1093/phr/116.5.404 PMC1497358 12042604

[29] Demsas F, Joiner MM, Telma K, Flores AM, Teklu S, Ross EG. Disparities in peripheral artery disease care: a review and call for action. Semin Vasc Surg. 2022. 10.1053/j.semvascsurg.2022.05.003. 10.1053/j.semvascsurg.2022.05.003 PMC9254894 35672104

[30] Havranek EP, Mujahid MS, Barr DA, Blair IV, Cohen MS, Cruz-Flores S, et al. Social determinants of risk and outcomes for cardiovascular disease: a scientific statement from the American Heart Association. Circulation. 2015. 10.1161/CIR.0000000000000228. 10.1161/CIR.0000000000000228 26240271

[31] Pande RL, Creager MA. Socioeconomic inequality and peripheral artery disease prevalence in US adults. Circ Cardiovasc Qual Outcomes. 2014. 10.1161/CIRCOUTCOMES.113.000618. 10.1161/CIRCOUTCOMES.113.000618 PMC4219271 24987053

[32] Hall WJ, Chapman MV, Lee KM, Merino YM, Thomas TW, Payne B, et al. Implicit racial/ethnic bias among health care professionals and its influence on health care outcomes: a systematic review. Am J Public Health. 2015. 10.2105/AJPH.2015.302903. 10.2105/AJPH.2015.302903 PMC4638275 26469668

[33] Gamble VN. Under the shadow of Tuskegee: African Americans and health care. Am J Public Health. 1997. 10.2105/ajph.87.11.1773. 10.2105/ajph.87.11.1773 PMC1381160 9366634

[34] Victor RG, Lynch K, Li N, Blyler C, Muhammad E, Handler J. A cluster-randomized trial of blood-pressure reduction in black barbershops. N Engl J Med. 2018. 10.1056/NEJMoa1717250. 10.1056/NEJMoa1717250 PMC6018053 29527973

[35] Sulaica EM, Wollen JT, Kotter J, Macaulay TE (2020). A review of hypertension management in black male patients. Mayo Clin Proc.

[36] Luque JS, Ross L, Gwede CK. Qualitative systematic review of barber-administered health education, promotion, screening and outreach programs in African-American communities. J Community Health. 2014. 10.1007/s10900-013-9744-3. 10.1007/s10900-013-9744-3 PMC3947222 23913106

[37] Linnan LA, D'Angelo H, Harrington CB (2014). A literature synthesis of health promotion research in salons and barbershops. Am J Prev Med.

[38] Palmer KNB, Rivers PS, Melton FL, McClelland DJ, Hatcher J, Marrero DG, et al. Health promotion interventions for African Americans delivered in U.S. barbershops and hair salons- a systematic review. BMC Public Health. 2021. 10.1186/s12889-021-11584-0. 10.1186/s12889-021-11584-0 PMC8365990 34399723

[39] Cutler JA, Sorlie PD, Wolz M, Thom T, Fields LE, Roccella EJ. Trends in hypertension prevalence, awareness, treatment, and control rates in United States adults between 1988-1994 and 1999-2004. Hypertension. 2008. 10.1161/HYPERTENSIONAHA.108.113357. 10.1161/HYPERTENSIONAHA.108.113357 18852389

[40] Gilbert KL, Ray R, Siddiqi A, Shetty S, Baker EA, Elder K, et al. Visible and invisible trends in black men's health: pitfalls and promises for addressing racial, ethnic, and gender inequities in health. Annu Rev Public Health. 2016. 10.1146/annurev-publhealth-032315-021556. 10.1146/annurev-publhealth-032315-021556 PMC6531286 26989830

[41] Alexander BK. Fading, twisting, and weaving: an interpretive ethnography of the black barbershop as cultural space. Qual Inq. 2003. 10.1177/1077800402239343.

[42] White Solaru K, Coy T, DeLozier S, Brinza E, Ravenell J, Longenecker C, et al. Findings of a novel barbershop-based peripheral artery disease screening program for black men. Journal of the American Heart Association. 2022. 10.1161/JAHA.122.026347. 10.1161/JAHA.122.026347 PMC9673675 36250671

[43] Aboyans V, Criqui MH, Abraham P, Allison MA, Creager MA, Diehm C, et al. Measurement and interpretation of the ankle-brachial index: a scientific statement from the American Heart Association. Circulation. 2012. 10.1161/CIR.0b013e318276fbcb. 10.1161/CIR.0b013e318276fbcb 23159553

[44] Griffith DM, Jaeger EC, Bergner EM, Stallings S, Wilkins CH. Determinants of trustworthiness to conduct medical research: findings from focus groups conducted with racially and ethnically diverse adults. J Gen Intern Med. 2020. 10.1007/s11606-020-05868-1. 10.1007/s11606-020-05868-1 PMC7573005 32495099

[45] Fryer CS, Passmore SR, Maietta RC, Petruzzelli J, Casper E, Brown NA, et al. The symbolic value and limitations of racial concordance in minority research engagement. SAGE Open. 2016. 10.1177/2158244020902082. 10.1177/1049732315575708 PMC4658313 25769299

[46] Randolph S, Coakley T, Shears J. Recruiting and engaging African-American men in health research. Nurs Res. 2018. 10.7748/nr.2018.e1569. 10.7748/nr.2018.e1569 29738190

[47] Dedoose Version, 0. 0. 17. Web application for managing, analyzing, and presenting qualitative and mixed method research data. 2021. www.dedoose.com.

[48] Braun V, Clarke V (2006). Using thematic analysis in psychology. Qual Res Psychol.

[49] Thomas DR. A general inductive approach for analyzing qualitative evaluation data. Am J Eval. 2006. 10.1177/1098214005283748.

[50] Attride-Stirling J. Thematic networks: an analytic tool for qualitative research. Qual Res. 2001. 10.1177/146879410100100307.

[51] Nowell LS, Norris JM, White DE, Moules NJ. Thematic analysis: striving to meet the trustworthiness criteria. Int J Qual Methods. 2017. 10.1177/1609406917733847.

[52] Carter N, Bryant-Lukosius D, DiCenso A, DiCenso A, Blythe J, Neville AJ. The use of triangulation in qualitative research. Oncol Nurs Forum. 2014. 10.1188/14.ONF.545-547. 10.1188/14.ONF.545-547 25158659

[53] Roth R, Barsi E (2005). The community need index. A new tool pinpoints health care disparities in communities throughout the nation. Health Prog.

[54] Whelton PK, Carey RM, Aronow WS, Casey DE Jr, Collins KJ, Dennison Himmelfarb C, et al. ACC/AHA/AAPA/ABC/ACPM/AGS/APhA/ASH/ASPC/NMA/PCNA guideline for the prevention, detection, evaluation, and management of high blood pressure in adults: a report of the American College of Cardiology/American Heart Association task force on clinical practice guidelines. J Am Coll Cardiol. 2017;2018. 10.1016/j.jacc.2017.11.006. 10.1016/j.jacc.2017.11.006 29146535

[55] Criqui MH, Matsushita K, Aboyans V, Hess CN, Hicks CW, Kwan TW, et al. Lower extremity peripheral artery disease: contemporary epidemiology, management gaps, and future directions: a scientific statement from the American Heart Association. Circulation. 2021. 10.1161/CIR.0000000000001005. 10.1161/CIR.0000000000001005 PMC9847212 34315230

[56] Victor RG, Ravenell JE, Freeman A, Leonard D, Bhat D, Shafiq M, et al. Effectiveness of a barber-based intervention for improving hypertension control in black men: the BARBER-1 study: a cluster randomized trial. Arch Intern Med. 2011. 10.1001/archinternmed.2010.390. 10.1001/archinternmed.2010.390 PMC3365537 20975012

[57] Osorio M, Ravenell JE, Sevick MA, Ararso Y, Young T, Wall SP, et al. Community-based hemoglobin A1C testing in barbershops to identify black men with undiagnosed diabetes. JAMA Intern Med. 2020. 10.1001/jamainternmed.2019.6867. 10.1001/jamainternmed.2019.6867 PMC6990850 31985740

[58] Rosenblatt AM, Crews DC, Powe NR, Zonderman AB, Evans MK, Tuot DS. Association between neighborhood social cohesion, awareness of chronic diseases, and participation in healthy behaviors in a community cohort. BMC Public Health. 2021. 10.1186/s12889-021-11633-8. 10.1186/s12889-021-11633-8 PMC8414876 34479522

[59] Moore N, Wright M, Gipson J, Jordan G, Harsh M, Reed D, et al. A survey of African American men in Chicago barbershops: implications for the effectiveness of the barbershop model in the health promotion of African American men. J Community Health. 2016. 10.1007/s10900-016-0152-3. 10.1007/s10900-016-0152-3 26831485

[60] Hammond WP, Matthews D, Mohottige D, Agyemang A, Corbie-Smith G. Masculinity, medical mistrust, and preventive health services delays among community-dwelling African-American men. J Gen Intern Med. 2010. 10.1007/s11606-010-1481-z. 10.1007/s11606-010-1481-z PMC2988156 20714819

[61] Cheatham CT, Barksdale DJ, Rodgers SG. Barriers to health care and health-seeking behaviors faced by black men. J Am Acad Nurse Pract. 2008. 10.1111/j.1745-7599.2008.00359.x. 10.1111/j.1745-7599.2008.00359.x 19128339

[62] Arnett MJ, Thorpe RJ Jr, Gaskin DJ, Bowie JV, LaVeist TA. Race, medical mistrust, and segregation in primary care as usual source of care: findings from the exploring health disparities in integrated communities study. J Urban Health. 2016. 10.1007/s11524-016-0054-9. 10.1007/s11524-016-0054-9 PMC4899337 27193595

[63] Hammond WP, Matthews D, Corbie-Smith G. Psychosocial factors associated with routine health examination scheduling and receipt among African American men. J Natl Med Assoc. 2010. 10.1016/S0027-9684(15)30600-3. 10.1016/s0027-9684(15)30600-3 PMC2865157 20437735

[64] Powell W, Adams LB, Cole-Lewis Y, Agyemang A, Upton RD. Masculinity and race-related factors as barriers to health help-seeking among African American men. Behav Med. 2016. 10.1080/08964289.2016.1165174. 10.1080/08964289.2016.1165174 PMC4979354 27337619

[65] Powell W, Richmond J, Mohottige D, Yen I, Joslyn A, Corbie-Smith G. Medical mistrust, racism, and delays in preventive health screening among African-American men. Behav Med. 2019. 10.1080/08964289.2019.1585327. 10.1080/08964289.2019.1585327 PMC8620213 31343960

