# Supplementary material for: Black men’s awareness of peripheral artery disease and acceptability of screening in barbershops: a qualitative analysis
Source: BMC Public Health. 2023 Jan 6;23:46. doi: 10.1186/s12889-022-14648-x (PMC9821364; doi:10.1186/s12889-022-14648-x)
Supplement: Supplementary file 1 — Additional file 1. HyPe The Cure Exit Interview Guide. [file 12889_2022_14648_MOESM1_ESM.docx]

**Additional File 1: HyPe The Cure Exit Interview Guide**

1.     How would you describe your overall experience in this study?

2.     As a part of this study, we had participants privately answer questions about themselves using an iPad instead of pen and paper or talking to a study team member. Did you find this method to be easy to use?

3.      Of the above 3 methods: (1) using the iPad to answer questions electronically (2) filling out surveys with pen and paper or (3) answering questions in person with a study team member, which would you prefer to use in a future study in the barbershop setting?

4.     Were you comfortable having your blood pressure checked in the arm while you were in the barbershop?

5.     Were you comfortable having your ABI done (blood pressure checked in arms and legs at same time while lying down) done in the barbershop?

6.     Did you experience any discomfort from having your blood drawn with the fingerstick test?

7.     Did you think that learning the results of your cholesterol or glucose blood test was useful to you?

8.     What would you improve about the barbershop ABI testing for PAD (blood pressure checked in arms and legs at same time while lying down)?

9.     Did you find the educational video easy to understand?

10.  What would you improve about the educational video?

11.  How did the blood pressure and ABI (blood pressure checked in arms and legs at same time while lying down) impact your attitude or behavior about your health, if at all? In other words, did knowing your blood pressure results or whether or not you had PAD impact how you think about your own health?

12.  How did the educational video impact your attitude or behavior about your health, if at all?

13.  Did you feel the compensation provided as a part of this study (haircut for each visit with $20 value, $20 [restaurant] gift card for watching the educational video) was appropriate, too much, or too little? Please tell us more about this.

14.  Did this study change your understanding of what PAD is?

15.  Did this study change your understanding of how PAD affects the black community?

16.  Has being in this study increased your willingness to follow up with a healthcare provider? If yes, what about this study has increased your willingness? If no, why not?

17.  What did you find burdensome or difficult about the following study procedures: Seated blood pressure measurements (hypertension screening)? ABI (PAD screening)? Fingerstick blood draws (cholesterol and glucose screening)? Completing surveys on iPad? Watching educational video?

18.  If given the opportunity, would you participate in this study again? If yes, why? If no, why not?
