# Supplementary material for: Black men’s awareness of peripheral artery disease and acceptability of screening in barbershops: a qualitative analysis
Source: BMC Public Health. 2023 Jan 6;23:46. doi: 10.1186/s12889-022-14648-x (PMC9821364; doi:10.1186/s12889-022-14648-x)
Supplement: Supplementary file 2 — Additional file 2. HyPe The Cure Final Adjudicated Codebook and Definitions. [file 12889_2022_14648_MOESM2_ESM.docx]

**Additional File 2: HyPe The Cure Final Adjudicated Codebook and Definitions**

| **Title** | **Description** |
| --- | --- |
| Barbershop | Barbershop as community - “Foot in the door” to healthcare |
| Community Health | The role of communities (social or geographic) in shaping health behavior.  - Descriptions of or insights to communities to which participants belong (e.g., “we often have work to make ends meet”)  - Expression of the desire to serve an altruistic role in the community  - Description of health or health habits of the community, either as a member (e.g., “we”) or descriptively (e.g., “they”) - Comments about the health of black community  - Influence communities have on health (i.e., promoting self-efficacy)  - Discussion of role models and their influence on health within the community |
| Healthcare | Perceptions of or experiences with the healthcare system and its effects on individual health or wellness.  - Propensity to seek healthcare - Personal or secondhand accounts of experiences with the healthcare system (i.e., barriers to care, negative/positive interactions)  - Philosophy of healthcare (“Why does healthcare exist?”) - Expressions of healthcare needs  - Personal accounts of how the interactions with the healthcare system have changed since participating in the study (e.g., changes to health-seeking behaviors, prompting conversations with providers about the study, etc.)  - Perceptions of healthcare from the lens of and specific to a black patient  - Comments as a Black patient on the treatment of, access to, or propensity to seek healthcare |
| Implementation | Perceptions of the implementation of the study, including personal experiences or perceived experiences of others with study procedures.   - Feedback or experiences (negative or positive) that may be useful to inform future studies.  - Comments about satisfaction with or recommendations about study procedures (e.g., ABI, blood draw) or recommended changes  - Comments about how compensation facilitated engagement with the study or health screenings in general  - Comments about using ease of use or challenges with technology (i.e., iPad) |
| Motivation to Participate in Study | Discussion or explanations of reasons participant decided to participate in the study, or reasons that would motivate participation in the future |
| Individual Health | Perceptions, attitudes, beliefs about current, past, or desired health status - Beliefs about personal health, including factors impacting health  - Discussion of health behaviors - Knowledge about personal health status (current or desired), e.g., blood pressure, family history, etc.)  Changes to lifestyle (either past, present, or future) that are directly tied to individual health or wellness  - Changes ascribed to engagement in the study  - Comments about participant self-efficacy to make change (e.g., change talk) |
| Knowledge | Comments about knowledge of information about chronic disease, health management, etc. - Reflections about how the study changed awareness or knowledge about HTN, PAD, or other health-related topic - Comments about desire to further learning - Expression of being reminded about prior knowledge (health, disease, etc.) - Comments about what may have stood in the way of increasing knowledge (e.g., medical jargon) |

*Note: Parent codes are highlighted in gray and associated child codes are below each parent code. “Barbershop” and “Community Health” are remaining child codes of the collapsed parent code, “Community.”*
