# Supplementary material for: Black men’s awareness of peripheral artery disease and acceptability of screening in barbershops: a qualitative analysis
Source: BMC Public Health. 2023 Jan 6;23:46. doi: 10.1186/s12889-022-14648-x (PMC9821364; doi:10.1186/s12889-022-14648-x)
Supplement: Supplementary file 3 — Additional file 3. HyPe The Cure Participant Community Needs Index (CNI)* Score by Zip Code. [file 12889_2022_14648_MOESM3_ESM.docx]

**Additional File 3: HyPe The Cure Participant Community Needs Index (CNI)* Score by Zip Code.**

| CNI Score | Number of Participants | Zip Code | Population |
| --- | --- | --- | --- |
| 5 | 1 | 44103 | 16415 |
| 5 | 4 | 44104 | 22169 |
| 5 | 7 | 44108 | 22759 |
| 5 | 3 | 44110 | 18395 |
| 4.8 | 1 | 44106 | 26807 |
| 4.8 | 1 | 44112 | 21052 |
| 4.2 | 2 | 44128 | 27529 |
| 4 | 2 | 44119 | 11782 |
| 3.6 | 1 | 44125 | 26858 |
| 3.4 | 3 | 44118 | 38835 |
| 3.4 | 1 | 44121 | 31493 |
| 2.6 | 1 | 44143 | 23874 |
| 2.4 | 1 | 44124 | 37756 |
|  | | | |

**The CNI maps socioeconomic factors, including income, education, education, and housing, to gauge the level of population health need for every zip code in the United States, with a score of 1.0 indicating the least need, and a score of 5.0 the most need compared to the US national average (score of 3.0).* ^12^
